# Reduction of ephrin-A5 aggravates disease progression in amyotrophic lateral sclerosis

**DOI:** 10.1186/s40478-019-0759-6

**Published:** 2019-07-12

**Authors:** Laura Rué, Patrick Oeckl, Mieke Timmers, Annette Lenaerts, Jasmijn van der Vos, Silke Smolders, Lindsay Poppe, Antina de Boer, Ludo Van Den Bosch, Philip Van Damme, Jochen H. Weishaupt, Albert C. Ludolph, Markus Otto, Wim Robberecht, Robin Lemmens

**Affiliations:** 1 Department of Neurosciences, Experimental Neurology and Leuven Brain Institute (LBI), KU Leuven – University of Leuven, Leuven, Belgium; 2 Laboratory of Neurobiology, Center for Brain & Disease Research, VIB, KU Leuven, Leuven, Belgium; 3 Department of Neurology, Ulm University Hospital, Ulm, Germany; 4 Department of Neurology, University Hospitals Leuven, Campus Gasthuisberg, Leuven, Belgium

**Keywords:** Amyotrophic lateral sclerosis, Neurodegeneration, Motor neuron, SOD1^G93A^, EphA4, Ephrin-A5

## Abstract

Amyotrophic lateral sclerosis (ALS) is a fatal neurodegenerative disease that affects motor neurons in the brainstem, spinal cord and motor cortex. ALS is characterized by genetic and clinical heterogeneity, suggesting the existence of genetic factors that modify the phenotypic expression of the disease. We previously identified the axonal guidance EphA4 receptor, member of the Eph-ephrin system, as an ALS disease-modifying factor. EphA4 genetic inhibition rescued the motor neuron phenotype in zebrafish and a rodent model of ALS. Preventing ligands from binding to the EphA4 receptor also successfully improved disease, suggesting a role for EphA4 ligands in ALS. One particular ligand, ephrin-A5, is upregulated in reactive astrocytes after acute neuronal injury and inhibits axonal regeneration. Moreover, it plays a role during development in the correct pathfinding of motor axons towards their target limb muscles. We hypothesized that a constitutive reduction of ephrin-A5 signalling would benefit disease progression in a rodent model for ALS. We discovered that in the spinal cord of control and symptomatic ALS mice ephrin-A5 was predominantly expressed in neurons. Surprisingly, reduction of ephrin-A5 levels in SOD1^G93A^ mice accelerated disease progression and reduced survival without affecting disease onset, motor neuron numbers or innervated neuromuscular junctions in symptomatic mice. These findings suggest ephrin-A5 as a modifier of disease progression that might play a role in the later stages of the disease. Similarly, we identified a more aggressive disease progression in patients with lower ephrin-A5 protein levels in the cerebrospinal fluid without modifying disease onset. In summary, we identified reduced expression of ephrin-A5 to accelerate disease progression in a mouse model of ALS as well as in humans. Combined with our previous findings on the role of EphA4 in ALS our current data suggests different contribution for various members of the Eph-ephrin system in the pathophysiology of a motor neuron disease.

**Electronic supplementary material:**

The online version of this article (10.1186/s40478-019-0759-6) contains supplementary material, which is available to authorized users.

## Introduction

Amyotrophic Lateral Sclerosis (ALS) is a neurodegenerative disease in which upper motor neurons in the motor cortex and lower motor neurons in the brainstem and spinal cord degenerate. The course of the disease is relentless and progressive and ultimately results in death 3–5 years after symptom onset [5]. A large number of mutations in a wide variety of genes can cause the disease, such as the copper–zinc superoxide dismutase (*Sod1*), chromosome 9 open reading frame 72 (*C9orf72*), fused in sarcoma (*Fus*) and TAR DNA-binding protein 43 (*Tardbp*) genes [39]. In ALS, there is not only diversity in the number of disease-causing genes, but also large differences exist in the phenotypic presentation of the disease even in patients carrying the same mutation [12, 36]. This indicates that other factors can alter disease onset and/or progression. We have previously shown that EphA4 is a disease modifier in ALS, and that reduction of its signalling ameliorates the motor phenotype in different ALS models [42]. EphA4 is a tyrosine kinase receptor member of the Eph/ephrin family, which is composed by Eph tyrosine kinase receptors and their ephrin ligands [34]. When receptor and ligand interact, bidirectional signalling occurs downstream of both Ephs and ephrins, giving rise to attractive or repulsive stimuli of two opposing cell membranes [34]. Although the role of EphA4 ligands in ALS is unknown, an EphA4 agonist that impedes EphA4 binding to its ligands, and an EphA4-Fc recombinant protein that reduces binding of EphA4 ligands to the receptor beneficially affected the phenotypes in mouse models for ALS [44, 47].

One particular EphA4 ligand, ephrin-A5 (efnA5), is of specific interest since its expression is increased in reactive astrocytes after stroke, resulting in reduced growth of cortical axonal projections during recovery [6, 23, 33]. Blocking efnA5 signalling improved the recovery of mice after stroke injury [33]. This upregulation of efnA5 in reactive astrocytes was not only induced after experimental stroke, but also by physical stress as shown in vitro [33], suggesting that efnA5 might be upregulated in astrocytes under stressful conditions. Since astrocytes, microglia and oligodendrocytes contribute to the selective vulnerability of motor neurons in ALS, the study of the role of efnA5 in astrocytes in this disease is of interest [1, 19, 45].

In vitro studies have shown that adding recombinant efnA5 or efnA5 overexpressing cells to neuronal cultures induces growth cone collapse and neurite retraction by affecting reorganization of the actin cytoskeleton [10, 11, 28, 29, 43]. During development, efnA5 is expressed in the ventral limb and also in developing motor axons that project from the lateral division of the lateral motor column (LMCl) in the spinal cord towards the limb. EfnA5 present in both compartments works simultaneously through forward and reverse signalling pathways that involve Eph receptors, to guide these axons so that they properly project dorsally in the limb [3, 27]. In ALS, denervation of neuromuscular junctions (NMJs) and axonal retraction occurs before motor neurons start degenerating, and compensatory re-sprouting mechanisms exist to re-innervate NMJs [14, 35]. Axonal guidance mechanisms involved during developmental stages might also play a role in disease mechanisms of de- and re-innervation.

Overall, given the role of efnA5 in astrocytes and in controlling attraction/repulsion responses, we aimed to investigate the expression and the role of efnA5 in a SOD1^G93A^ mouse model of ALS. We hypothesized efnA5 reduction to improve disease progression in a mouse model for ALS, potentially via a role in the communication between astrocytes and motor neurons, and/or in axonal retraction and re-sprouting.

## Materials and methods

### Animals

All animal experiments were carried out in accordance with the U.K. Animals (Scientific Procedures) Act, 1986 and associated guidelines, EU Directive 2010/63/EU for animal experiments, and all animal experiments were approved by the local ethical committee of the KU Leuven (P229/2013 and P229/2017). Animals were housed under standard conditions according to the guidelines of the University of Leuven (KU Leuven), with a 12 h light-dark cycle and with access to food and water ad libitum. The human wild-type SOD1 overexpressing mouse (B6.SOD1^WT^; B6SJL-Tg(SOD1)2Gur/J; stock number 002297) and the human mutant SOD1 overexpressing mouse (B6.SOD1^G93A^; B6.Cg-Tg(SOD1*G93A)1Gur/J; stock number 004435) were purchased from the Jackson Laboratory (Ben Harbor, ME), and maintained in a C57BL/6 J genetic background. The efnA5 knockout mouse colony was generated by Dr. Jonas Frisén (Karolinska Institute, Stockholm, Sweden) [16], and was kindly provided by Dr. Renping Zhou (Rutgers University, Piscataway, NJ, USA). Mice were received and maintained under a mixed C57BL/6 J and S129 background, since EfnA5^−/−^ mice are embryonically lethal in a pure C57BL/6 J background [38]. For ALS-related experimental purposes, EfnA5^+/−^ mice were backcrossed for more than 10 generations to obtain EfnA5^+/−^ mice in a pure C57BL/6 J background, which were crossed with SOD1^G93A^ mice to obtain the following experimental groups: SOD1^G93A^ EfnA5^+/+^ and SOD1^G93A^ EfnA5^+/−^ mice. Only the mice whose father had a lifespan shorter than 165 days, were used for breeding and for experimental purposes. Only litters that contained the genotype of interest and the control genotype within the same gender were included in the experiments. Both males and females were used, and all experiments were conducted in a blinded manner.

### Animal motor performance

Established guidelines were followed to assess ALS disease phenotype [26]. The ability of mice to hang upside down from an elevated grid (hanging wire test) and their ability to walk on the rotarod rotating at a fixed speed of 15 rpm, was measured to determine motor coordination and strength. The latency to fall was measured with cut-offs of 60 s in the hanging wire test and of 300 s in the rotarod. Motor performance on these tests and weight were assessed in experimental mice three times every week from the age of 60 days until an end-stage point of the disease. Mice reached a disease end-stage point when their righting reflex took longer than 10 s. According to humane-endpoints, mice were euthanized at this time point and the date was annotated for survival analysis. Disease onset was determined as the age at which mice could no longer perform the maximum score of 60 s in the hanging wire test, and disease duration was calculated as the time between disease onset and mouse survival.

### Nerve conduction studies

Recordings of the compound muscle action potential (CMAP) were performed once a week in mice from the age of 60 days until the age of 115 days. Mice were anesthetized with 3% isoflurane/O_2_ gas inhalation and immobilized on a heating pad at 37 °C. Recordings were performed with subdermal needle electrodes (Technomed Europe). The stimulating electrode was placed at the level of the sciatic notch and the recording electrode was placed on the gastrocnemius muscle. CMAPs were measured by supramaximal stimulation (1 pulse per second and 0.1 ms stimulus duration) using a Medelec EMG monitor (Medelec Vickers) and Synergy software (version 20.1.0.100).

### Motor neuron counts and neuromuscular junctions in ALS mice

ALS mice were followed with hanging wire test, and a whole litter was sacrificed as soon as one mouse of the litter could no longer hang from the wire for 2 s, which was considered as a late-symptomatic stage of the disease [42]. At that point, an overdose of Dolethal (20 mg/ml; Vetoquinol) was used to sacrifice the litter. Gastrocnemius muscles were dissected and snap-frozen in ice-cold isopentane, and then mice were transcardially perfused with 4% paraformaldehyde (PFA). Lumbar spinal cords were dissected, post-fixed overnight with 4% PFA, cryoprotected with 30% sucrose and embedded in OCT embedding matrix (CellPath) and stored at − 80 °C for later use. For motor neuron counts, spinal cords were cryosected on a CryoStar NX70 Cryostat (ThermoFischer Scientific). Every sixth 20 μm-thick cryosection was stained with Neurotrace 500/525 (1:100; N21480; ThermoFischer) or cresyl violet (Sigma) and used for quantitative analysis of the number of neurons. Images of ten sections in total per spinal cord were obtained with a Zeiss Axioimager M1 epifluorescence and brightfield upright microscope with a Zeiss A-Plan 10X/0.25 objective (Carl Zeiss, Germany), and an Axiocam MRm monochrome digital camera and the Zen 2.3 software (Carl Zeiss, Germany). Only neurons within one ventral horn of each spinal cord were counted using ImageJ v1.51u by Wayne Rasband (National Institutes of Health) and the number of neurons in different size groups was determined. To quantify the innervation of NMJs, longitudinal 20 μm-thick gastrocnemius cryosections were stained overnight at 4 °C with Alexa-488-conjugated neurofilament-L antibody (1:500; Cell Signaling Technologies) and 1 h at RT with Alexa-555-conjugated α-bungarotoxin (Invitrogen; 1:1000). Slides were mounted with ProLong Gold antifade reagent (Life Technologies), and visualized with a Zeiss Axioimager M1 epifluorescence upright microscope with a Zeiss A-Plan 40X/0.65 ∞/0.17 objective (Carl Zeiss). At least 100 NMJs per muscle were analysed for innervation, as determined by the co-localization of the neurofilament-L and α-bungarotoxin labelling. Analyses were performed in a blinded manner. Representative Z-stack confocal images were obtained on a Leica TCS SP8 confocal laser scanning microscope (Leica Microsystems Heidelberg GmbH) with an HC PL APO CS2 40x/0.85 dry lens with a pinhole of 0.5 Airy Units. Sum projections of the Z-stacks was done with the freeware ImageJ v1.51u by Wayne Rasband (National Institutes of Health).

### Sciatic nerve crush surgery

Four months-old EfnA5^+/+^ and EfnA5^−/−^ mice maintained under a mixed C57BL/6 J and S129 background were deeply anesthetized for surgery with 3% isoflurane/O_2_ gas inhalation. Vetergesic (15 mg/kg; Ecuphar) was administered subcutaneously to minimize post-operative pain. The fascial plane between the gluteus maximus and the anterior head of the biceps femoris was opened in both hind limbs to expose the sciatic nerves, but while the left one was crushed, the right one was left untouched. The crush was performed at 42 mm from the most distal toe three times during 15 s with Dumont #5/45 Forceps (F.S.T.). Graphite powder (Sigma-Aldrich) was used to mark the crush site at the third crush. Finally, musculature and skin were sutured with non-absorbable ethicon-coated vicryl 6–0 sutures (Johnson & Johnson) and mice were allowed to recover under a heating lamp. Mice were sacrificed with an overdose of Dolethal (20 mg/ml; Vetoquinol) 11 days after the surgery. Gastrocnemius and tibialis anterior muscles were dissected and snap-frozen in ice-cold isopentane. Innervation of NMJs was assessed as for the ALS mice.

### RNAscope *in situ* hybridization

An overdose of Dolethal (20 mg/ml; Vetoquinol) was used to sacrifice ALS mice at 135 days of age. Mice were transcardially perfused with 4% paraformaldehyde (PFA). Spinal cords were then dissected, post-fixed overnight with 4% PFA, cryoprotected with 30% sucrose and embedded in OCT embedding matrix (CellPath) and stored at − 80 °C for later use. A CryoStar NX70 Cryostat (ThermoFischer Scientific) was used to cut 20 μm-thick cryosections, which were mounted on Superfrost Plus slides (ThermoFischer Scientific). To improve spinal cord slice attachment on the slides, slices were post-fixed during 30 min with cold 4% PFA (Life Technologies) at 4 °C, rinsed with PBS (Sigma-Aldrich), dehydrated with sequential incubation steps of 50, 70 and 100% ethanol, and baked for 30 min at 60 °C. RNAscope in situ hybridization was performed as indicated by the manufacturer with the RNAscope Multiplex Fluorescent Reagent Kit v2 (ACD Diagnostics). In brief, slides underwent an antigen retrieval step of 5 min at 98-104 °C with a Braun Multiquick FS-3000 Steamer (Braun) and a Protease III incubation step of 30 min at 40 °C in a HybEZ™ Oven (ACD Diagnostics). RNAscope probes against *Efna5* (RNAscope Probe - Mm-Efna5-C1; ACD Diagnostics), *Synaptophysin* (*Syp*; 1:100; RNAscope Probe - Mm-Syp-C3; ACD Diagnostics) and *Slc1a3* (1:50; RNAscope Probe - Mm-Slc1a3-C2) were used. Signal amplification was performed as stated in the manufacturer’s instructions with TSA Plus Fluorescein (1:750; Perkin Elmer), TSA Plus Cyanine 3 (1:500; Perkin Elmer) and TSA Plus Cyanine 5 (1:3000; Perkin Elmer). Nuclear counterstain was performed with Hoechst 33342 (5 μg/ml; Sigma) and slides were finally mounted with ProLong Gold antifade reagent (Life Technologies). Images of the ventral horns of every spinal cord section were obtained with a Leica TCS SP8 confocal laser scanning microscope (Leica Microsystems Heidelberg GmbH, Manheim, Germany) with an HC PL APO CS2 20x/0.75 dry lens and a pinhole of 0.5 Airy Units. Sum projection of 2 Z-stacks separated 2 μm from each other was done with the freeware ImageJ v1.51u by Wayne Rasband (National Institutes of Health) and images were next automatically quantified with the NIS-Elements Microscope Imaging Software (Nikon). Cells positive for *Syp* were detected based on intensity and designated as neurons. All detected nuclei that did not colocalize with *Syp*-positive cells were considered as glial nuclei, and a small perimeter (NIS-Elements thickening command, three iterations) around each nucleus was also selected to detect part of the cytoplasm of these cells. Glial nuclei with the small perimeter around them were considered as glial cells. Neurons were subdivided into large and small neurons according to the *Syp*-positive area: > 400 μm^2^ and 150–400 μm^2^, respectively, as previously described [15, 22, 42]. In accordance with Lalancette-Herbert et al., the cut-off of 400 μm^2^ used in our study differentiates alpha-motor neurons from gamma-motor neurons [22]. Although atrophy of alpha-motor neurons is present in SOD1^G93A^ mice, such cut-off still allows differentiating both motor neuron subtypes in this mouse model [22]. *Slc1a3* was used as an astrocyte marker. Neurons and glial cells containing at least two *EfnA5* puncta or three *Slc1a3* puncta were considered *EfnA5* and/or *Slc1a3* positive. Single-cell *EfnA5* expression levels, were calculated as dot density in every cell analysed.

### RNA extraction, quantitative PCR and digital droplet PCR

ALS mice were sacrificed at 130 days of age or at end-stage point of the disease with cervical dislocation and lumbar spinal cords were dissected and homogenized in TRIzol (ThermoFischer Scientific) with Lysing Matrix D beads (MP Biomedicals) and a MagNa Lyser oscillator (Roche) at 6500 rpm for 30 s thrice with a 1 min interval on ice. Total RNA was precipitated with isopropanol. cDNA was prepared with the SuperScript III First-Strand Synthesis System (ThermoFischer Scientific). Quantitative PCRs were performed with the TaqMan Fast Universal PCR Master Mix 2X (Life Technologies) and the following Taqman assays: *Chat* (Mm01221882_m1), *Rbfox3* (Mm01248771_m1) and *Aif* (Mm00479862_g1) from Life technologies, and *Efna5* (Mm.PT.58.28681125), *Gfap* (Mm.PT.58.10570926), *Vimentin* (Mm.PT.58.8720419), *Polr2a* (Mm.PT.58.13811327) and *Gapdh* (Mm.PT.39a.1) from IDT. Reaction was performed in a StepOnePlus instrument (Life Technologies) and relative gene expression was analysed with the Qbase+ software (Biogazelle). Digital droplet PCR was performed following Bio-Rad guidelines. PCR was done with ddPCR Supermix for Probes (No dUTPs; Bio-Rad) and with *Efna5*-FAM Taqman assays Mm.PT.58.28681125 (exon 1–2) and Mm.PT.58.29725953 (exon 3–5) from IDT, multiplexing each of them with *Polr2a*-HEX labelled Taqman assay Mm.PT.58.13811327 (Integrated DNA Technologies). Droplets were generated in a QX200 Droplet Generator and scanned with a QX200 Droplet Reader (Bio-Rad). Results were analysed with QuantaSoft software from Bio-Rad.

### Label-free proteomic analysis of tissue samples

Lumbar spinal cord samples were obtained from SOD1^WT^, SOD1^G93A^ EfnA5^+/+^ and SOD1^G93A^ EfnA5^+/−^ mice at 130 days of age after cervical dislocation, and were lysed by addition of lysis buffer containing 6 M guanidine hydrochloride, 10 mM Tris (2-carboxyethyl)phosphine hydrochloride (TCEP), 40 mM 2-chloroacetamide and 100 mM triethylammonium bicarbonate (TEAB). Samples were then sonicated, heated at 95 °C for 10 min and centrifuged at 20000 g for 30 min at 4 °C, and the supernatant was collected for further analysis. Protein concentration was determined using the Qubit Fluorometric Quantification Kit (Thermo Scientific). Protein digestion was performed with a filter-aided sample preparation (FASP) protocol and analysed by LC-MS/MS as previously described [4]. Proteins were identified using MaxQuant 1.5.2.8 and the *Mus musculus* reference proteome from UniProt (downloaded 12th March 2018). A false discovery rate (FDR) of 1% was used for peptide and protein identification (total protein IDs after exclusion of contaminants: 5525) and protein quantification was performed with the MaxLFQ algorithm [8]. Quantitative analysis of the data was performed with Perseus 1.5.2.6 [40]. Proteins with valid values in at least three samples per group were used for the quantitative comparison (4065 proteins).

### Patients and cerebrospinal fluid collection

Patients (Table 1) were enrolled at the Ulm University Hospital, Department of Neurology, where cerebrospinal fluid (CSF) was collected by lumbar puncture at diagnostic follow-up (except asymptomatic ALS gene carriers and non-carriers, see below). Samples were centrifuged and stored at − 80 °C within 2 h according to standard operating procedures. Control patients had no neurodegenerative disease and CSF was collected to rule out acute or chronic inflammation of the brain. Asymptomatic ALS gene carriers and their relatives without known ALS mutation were recruited via the German Presymptomatic-ALS cohort [32]. ALS was diagnosed according to Ludolph et al. [25] and disease onset was determined by interviewing of the patients. All patients or their relatives gave written informed consent to participate in the study, and the collection and analysis of CSF samples during the diagnostic pathway was approved by the Ethics Committee of Ulm University.

**Table 1:** Table showing the characteristics of the humansamples analysed in the present study. The total number (N) ofsamples and the median age with its interquartile range areshown for every group: Young and aged controls (Con),asymptomatic ALS patients (Asympt.), patients carrying a knowngenetic mutation (gALS) and patients without any identifiedgenetic mutation (sALS)

	Con (young)	Con (aged)	Asympt. ALS	gALS	sALS
N (f/m)	24 (14/10)	32 (13/19)	21 (14/7)	38 (14/24)	70 (25/45)
Age (yr)	42 (31-50)	63 (57-72)	45 (35-51)	59 (51-67)	65 (56-70)

### Multiple reaction monitoring of CSF samples

For multiple reaction monitoring (MRM) analysis of efnA5 in CSF samples, 200 μL of CSF were spiked with TEAB buffer and a quantitative protein epitope signature tag (QPrEST, Atlas Antibodies AB, #QPrEST25042) of efnA5 as internal standard. Samples were reduced and alkylated with 1 mM TCEP and 1 mM CAA at 95 °C for 10 min. Proteins were then digested for 16 h at 37 °C by adding 1.2 μg trypsin/LysC (Promega). Digestion was stopped by addition of 800 μL 1.25% TFA and peptides were transferred to strong cation exchange STAGE-Tips by centrifugation as previously described [31]. Peptides were washed with 0.2% TFA and fractionated with 75, 125, 200, 300, 450 mM ammonium acetate in 20% acetonitrile/0.5% formic acid (fraction 1–5) and 5% ammonium hydroxide/80% acetonitrile (fraction 6). After vacuum drying, peptides were dissolved in 27.5 μL 6% acetonitrile/0.1%TFA and analysed by LC-MRM.

Analysis of efnA5 was performed with a QTRAP6500 mass spectrometer (AB Sciex), Eksigent MicroLC200 and Agilent 1260 HPLC pump. The fractions were loaded on a C18 PepMap100, 5 μm, 0.3 × 5 mm trap column (Thermo Fisher Scientific). Separation was performed on an Eksigent HALO Fused-core C18, 2.7 μm, 0.5 × 100 mm column at 40 °C with mobile phase A: 4% DMSO/0.1% formic acid, and mobile phase B: 4% DMSO/96% acetonitrile/0.1% formic acid and a linear gradient from 1 to 30% B within 9.85 min. The following transitions of efnA5 (peptide TIGVHDR) were measured: 399.2–583.3 (y5), 399.2–427.2 (y3) (light peptide); 404.2–593.3 (y5), 404.2–437.2 (y3) (heavy peptide). For relative quantification, the light-to-heavy (L/H) peptide ratio (mean of the two transitions) was calculated using Skyline v4.2. CSF quality control (QC) samples were included in each run.

### Statistics

Statistics were performed using Graphpad prism 7.01 software (Graphad Software), except for the analysis of the proteomic data (see below). Unpaired two-tailed t-test, one-way ANOVA followed by the Dunnett’s multiple comparisons posthoc test, two-way ANOVA with repeated measurements followed by the Sidak’s multiple comparisons posthoc test and two-way ANOVA followed by the Tukey’s multiple comparisons posthoc test, were used to determine differences among conditions as stated in the figure legends. Survival and disease onset were analysed using the Log-rank test if data showed a parametric distribution. For non-parametric data, a two-tailed Mann-Whitney test was performed. Statistical analysis of proteomic data was performed with Perseus 1.5.2.6. Protein levels between groups were compared by two-tailed Student’s t-test applying an FDR of 5% and S0 = 0.1 to correct for multiple testing. Data are visualized by Volcano plots. For efnA5 protein determination in CSF samples, groups were compared by Kruskal-Wallis test and Dunn’s post hoc test. A *p*-value < 0.05 was regarded as significant. For all the tests, significance level was considered for *p* values lower than 0.05.

Based on previous experiments in SOD1^G93A^ mice, we estimated that a sample size of 32 mice in each group would provide 80% power at an α = 0.05 to detect an extended survival of 11 days, as previously found for EphA4^+/−^ SOD1^G93A^ mice [42]. For the sciatic nerve crush experiments, we estimated a sample size of 4–6 mice per group to have an 80% power at α = 0.05 to detect a difference of 24% in re-innervation as previously observed for EphA4^−/−^ mice [42]. For these experiments we used 5 mice per group.

## Results

### Ephrin-A5 is mainly expressed in neurons of the ventral horn

**Fig. 1:**
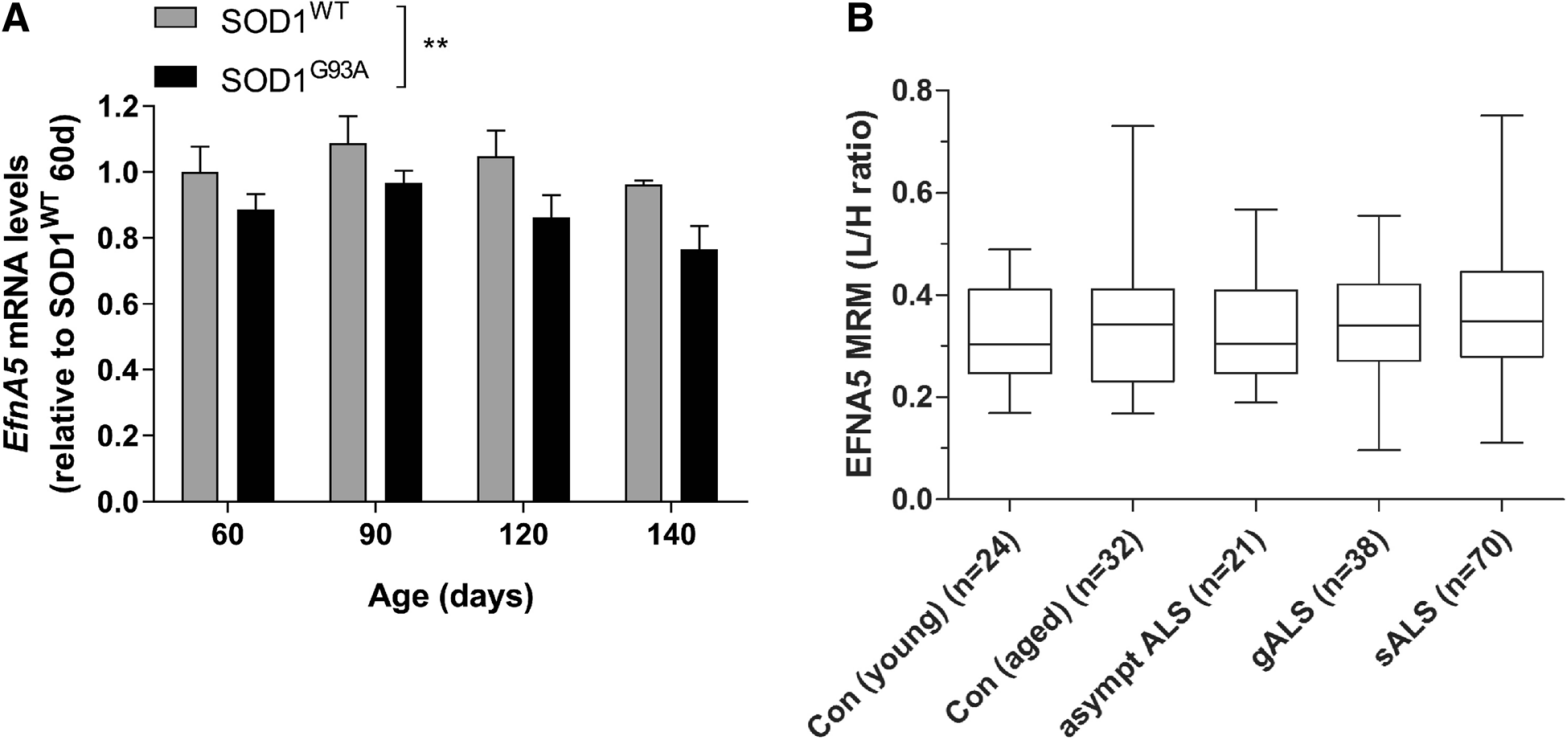
*EfnA5* gene expression levels are reduced in SOD1^G93A^ mice whereas efnA5 protein is not altered in ALS patients CSF. **a** *EfnA5* gene expression was determined in the lumbar spinal cord of SOD1^WT^ and SOD1^G93A^ overexpressing mice (*N* = 4–9). Data represents mean ± SEM, and different conditions were compared with a two-way ANOVA with Tukey’s multiple comparisons test: ** *P* < 0.01 as compared with SOD1^WT^. **b** EfnA5 protein relative quantification in the CSF of patients carrying an ALS genetic known mutation (gALS) and patients without any known mutation (sALS) were compared to the levels found in the CSF of young and aged controls (Con) and asymptomatic ALS patients carrying a known mutation for the disease (asympt ALS). Boxes show median and interquartile range, and whiskers are minimum and maximum. The light-to-heavy (L/H) peptide ratio was used as relative quantification of EfnA5 protein

We first determined *EfnA5* gene expression in whole lysates of adult mice lumbar spinal cord in age-matched SOD1^WT^ and mutant SOD1^G93A^ mice, at different disease stages (pre-symptomatic, onset, and early and late-symptomatic). *EfnA5* mRNA levels were lower in mutant SOD1^G93A^ mice compared to SOD1^WT^ (Fig. 1a). We also quantified the amount of efnA5 protein with MRM in CSF from ALS patients and controls. No difference in the protein amount was detected when we compared aged and young control samples, and no differences were observed when we compared control, non-symptomatic and symptomatic ALS samples (Fig. 1b).

**Fig. 2:**
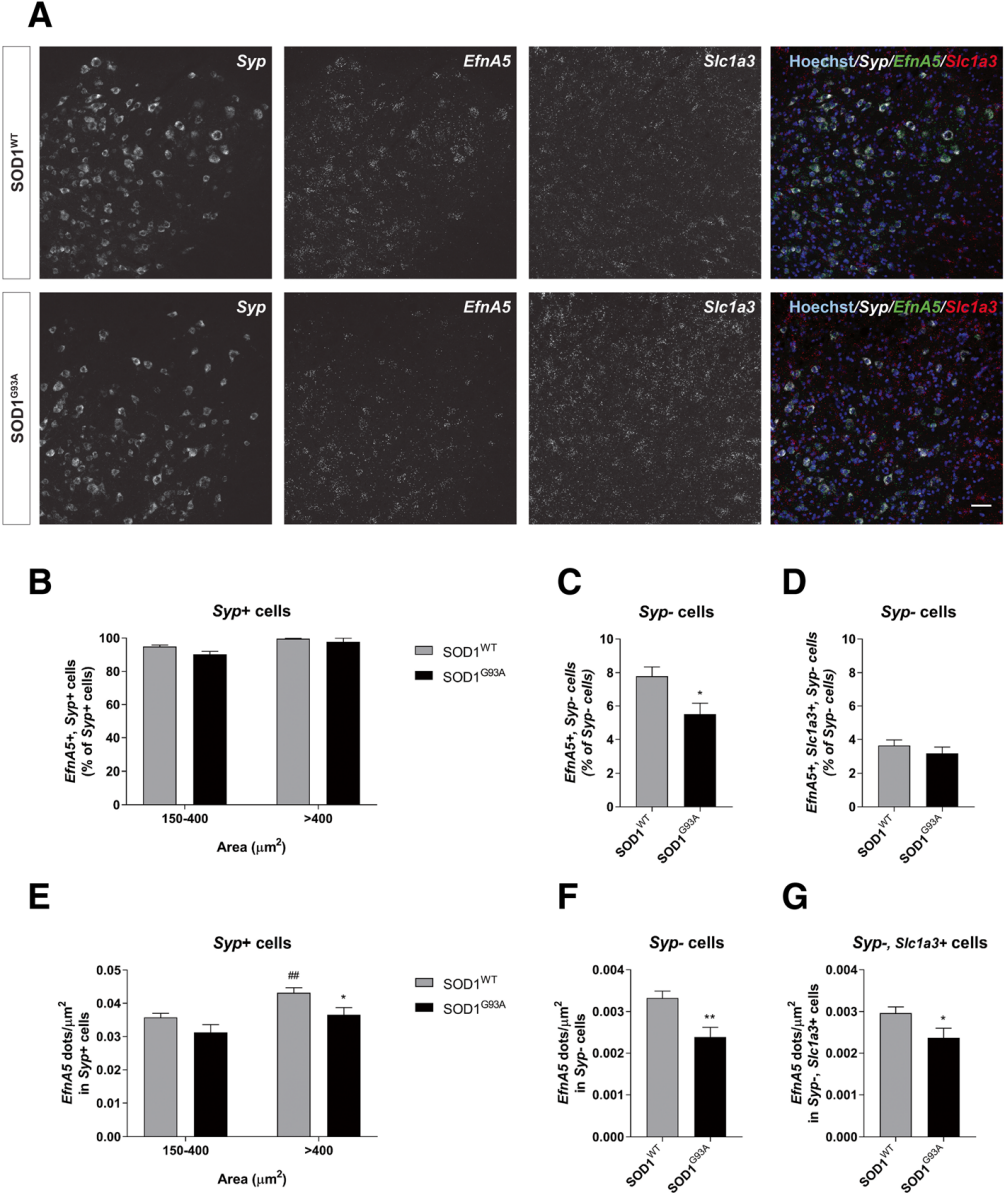
In the ventral horn of the spinal cord *EfnA5* is mainly expressed in neurons. RNAscope in situ hybridization was performed to determine cell type-specific expression of *EfnA5*. A total of 15–18 images of different lumbar spinal cord ventral horns from 3 different mice were analysed for every condition. **a** Representative images show lumbar spinal cord ventral horns of SOD1^WT^ and SOD1^G93A^ mice at 135 days of age that were stained with probes against *EfnA5, Syp* and *Slc1a3*. Hoechst was used as a counter stain for nuclei. Scale bar = 50 μm. **b** Percentage of neurons (*Syp* + cells) that stained positive for *EfnA5* were quantified and shown as percentage of the total *Syp* + cells. **c** Glial cells (*Syp*- cells) and **d** astrocytes (*Syp*-, *Slc1a3*+ cells) positive for *EfnA5* were quantified and represented as percentage of the total amount of glial cells. Single-cell *EfnA5* expression was quantified by measuring *EfnA5* puncta density (dots/μm^2^) in **e** neurons, **f** glial cells and **g** astrocytes. Data represents mean ± SEM, and different conditions were compared with a two-tailed t-test: * *P* < 0.05; ** *P* < 0.01 as compared to SOD1^WT^. **b** and **e** Data represents mean ± SEM, and different conditions were compared with a two-way ANOVA with Tukey’s multiple comparisons test: * *P* < 0.05 as compared with SOD1^WT^; ^##^ *P* < 0.01 as compared with 150–400 μm^2^ neurons

To determine cell type specific expression of efnA5 and potential single-cell changes related to ALS, we performed RNAscope in situ hybridizations in lumbar spinal cord slices of SOD1^WT^ and SOD1^G93A^ symptomatic mice at 135 days of age and examined *EfnA5* gene expression in the ventral horn in neurons and in glial cells (Fig. 2a). Since in ALS different vulnerability has been observed in neurons of different soma size [42], *EfnA5* expression was evaluated in the most vulnerable, large, (> 400 μm^2^) and small (150–400 μm^2^) neurons. In SOD1^WT^ spinal cords, more than 92% of the neurons (*Syp* positive cells) expressed *EfnA5* (Fig. 2b). In contrast, only 8% of glial cells (*Syp* negative cells) and 4% of astrocytes (*Syp* negative, *Slc1a3* positive cells) expressed *EfnA5*, indicating that in the spinal cord *EfnA5* is mainly expressed in neurons and minimally in astrocytes (Fig. 2c, d). In the SOD1^G93A^ spinal cord the percentage of *EfnA5* positive large and small neurons and astrocytes was similar to SOD1^WT^, but we observed a reduction of 2% of total glial cells expressing *EfnA5* (Fig. 2b-d). We next determined differential expression of *EfnA5* in neurons and glia at a single cell level. *EfnA5* expression was higher in the large compared to the small neurons in SOD1^WT^ mice (Fig. 2e), and we observed a reduction of *EfnA5* in individual large neurons of SOD1^G93A^ compared to SOD1^WT^ mice (Fig. 2e), as well as in glial cells including astrocytes (Fig. 2f, g).

### Reduction of ephrin-A5 levels accelerates disease progression in SOD1^G93A^ mice

**Fig. 3:**
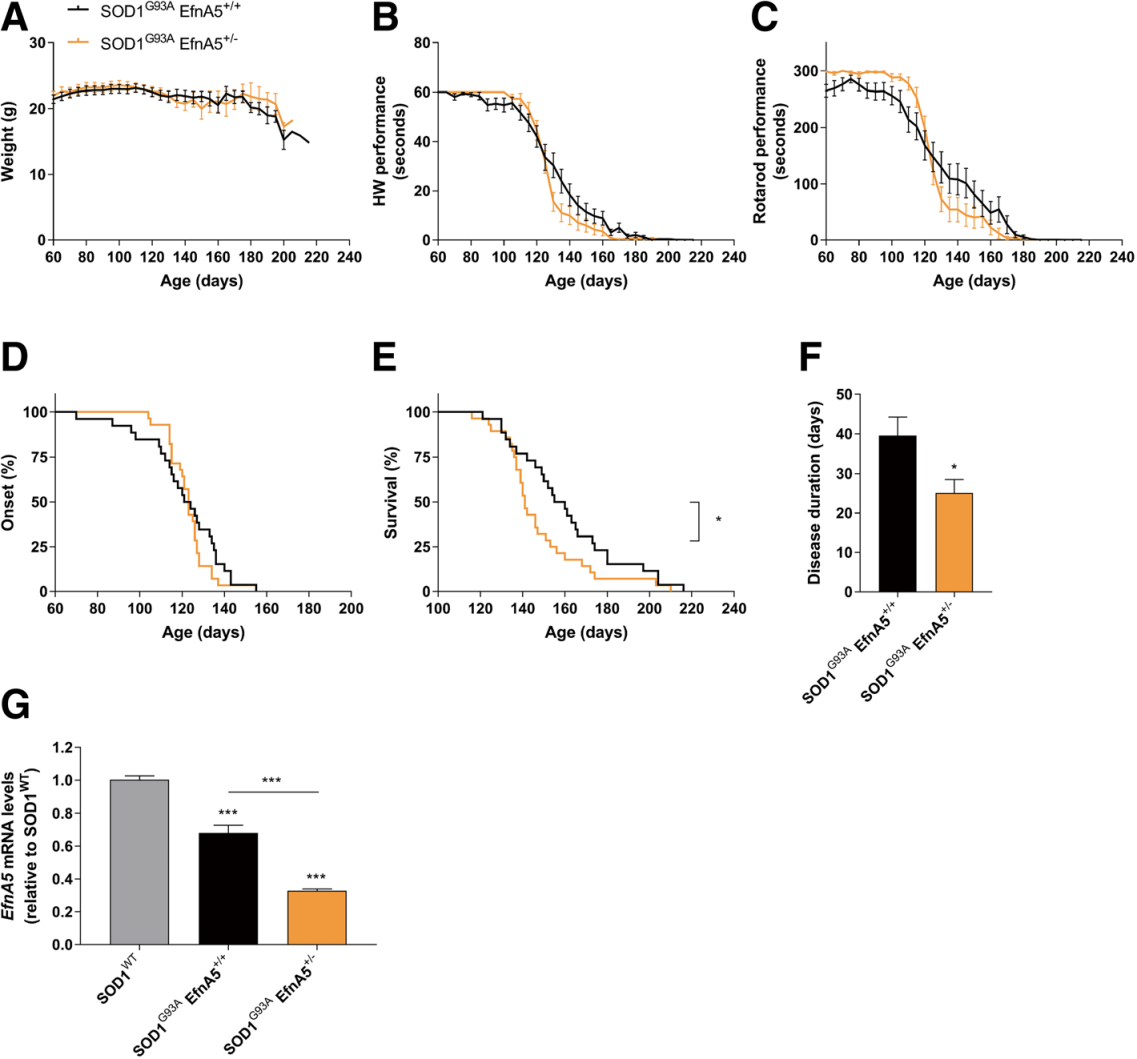
Reduction of efnA5 levels in SOD1^G93A^ mice is detrimental and shortens survival and disease duration. Disease progression was closely monitored in **a**-**f** SOD1^G93A^ EfnA5^+/+^ and SOD1^G93A^ EfnA5^+/−^ mice from the age of 60 days onwards (*N* = 26–28). **a** Weight and **b** and **c** motor performance decline over time on **b** the hanging wire (HW) and **c** rotarod tests is shown. Data is represented as mean ± SEM and two-way ANOVA test with repeated measurements was used to determine differences among genotypes. **d** Disease onset differences were compared between the two genotypes with the Log-rank test whereas **e** survival was analysed with the two-tailed Mann-Whitney test for non-parametric data: * *P* < 0.05. **f** Disease duration was analysed with a two-tailed student t-test: * *P* < 0.05. **g** *EfnA5* expression levels in the lumbar spinal cord of end-stage mice were determined by qPCR and data is represented as mean ± SEM (*N* = 7–9). Different genotypes were compared with a one-way ANOVA with Tukey’s multiple comparisons test: *** *P* < 0.001

In contrast to a stroke model [33], *EfnA5* was minimally expressed in astrocytes. Similarly to EphA4 [42], *EfnA5* expression was higher in the large and most vulnerable motor neurons, and therefore, we hypothesized that this higher expression could contribute to mechanisms that drive to the selective retraction and vulnerability of large motor neurons in ALS [14, 35]. To study disease progression in SOD1^G93A^ mice with lower efnA5, we reduced *EfnA5* expression by crossbreeding SOD1^G93A^ mice with a constitutive EfnA5 knockout mouse [16]. Neurodevelopmental axon miswiring alterations have been reported in EfnA5^−/−^ mice [2, 9, 16, 21], but EfnA5^+/−^ and EfnA5^−/−^ adult mice had similar amounts of motor neurons in the ventral horn of the lumbar spinal cord and innervated NMJs in the gastrocnemius muscle as compared to EfnA5^+/+^ mice (Additional file 1: Figure S1). Since EfnA5^−/−^ mice present with embryonic lethality when they are bred in a C57BL/6 J background [38], we crossbred SOD1^G93A^ mice to EfnA5^+/−^ mice and thus reduced efnA5 levels to 50%. SOD1^G93A^ EfnA5^+/−^ mice had comparable body weight, as assessed from 60 days until mice reached an end-stage point of the disease (Fig. 3a). Disease onset was unaltered and motor performance in both hanging wire and rotarod tests of SOD1^G93A^ EfnA5^+/−^ mice progressively declined in a similar manner as SOD1^G93A^ EfnA5^+/+^ mice (Fig. 3b-d). However, mice reached disease end-stage faster than their littermate controls, with a median survival reduction of 17 days (Fig. 3e), which was also reflected in a shorter disease duration (25 days in SOD1^G93A^ EfnA5^+/−^ compared to 40 days in SOD1^G93A^ EfnA5^+/+^) (Fig. 3f). We excluded compensatory mechanisms potentially resulting in upregulation of *EfnA5* gene expression by confirming a 50% reduction in *EfnA5* expression levels at end-stage in SOD1^G93A^ EfnA5^+/−^ compared to SOD1^G93A^ EfnA5^+/+^ (Fig. 3g). Since we observed an accelerated disease progression instead of an extended survival, we stopped the experiment with 26 and 28 mice per group.

### Reduction of ephrin-A5 levels does not alter electrophysiological and histological findings at a symptomatic stage of the disease

**Fig. 4:**
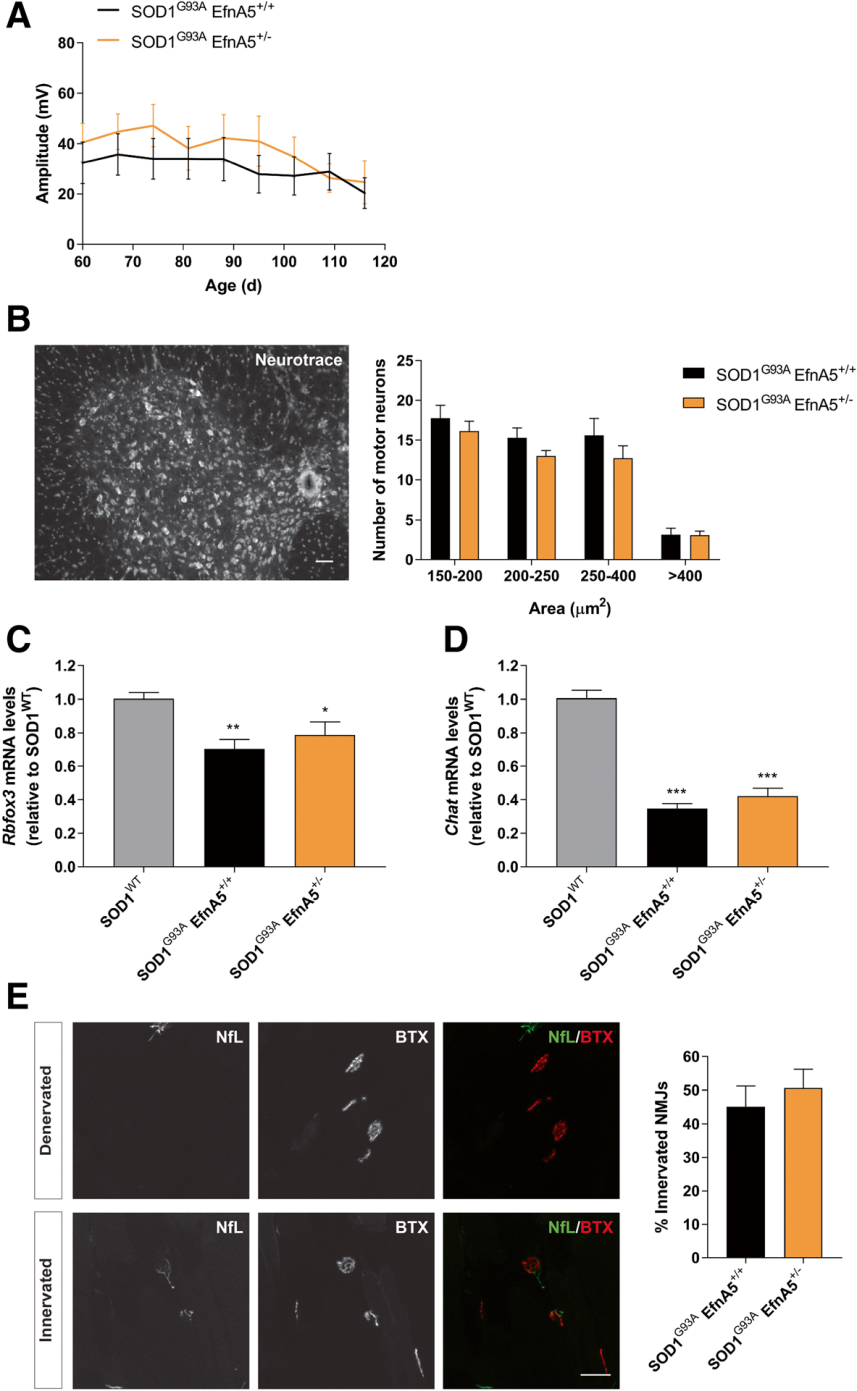
Electromyography decline, neuronal loss and neuromuscular junction denervation in SOD1^G93A^ mice is not affected in SOD1^G93A^ mice with reduced efnA5 levels. **a** Compound muscle action potential amplitude (expressed in mV) was measured in SOD1^G93A^ EfnA5^+/+^ and SOD1^G93A^ EfnA5^+/−^ mice from the age of 60 days (d) onwards. Data is represented as as mean ± SEM (*N* = 7–8). **b** The number of motor neurons of different soma area was counted in the ventral horn of the lumbar spinal cord. Data is represented as mean ± SEM (*N* = 6–7) and it was analysed with a two-way ANOVA. Representative image shows Neurotrace staining of a spinal cord slice. Scale bar = 50 μm. **c** and **d** Expression levels of **c** *Rbfox3* and **d** *Chat* were unaltered after efnA5 knockdown in mice at 130 days of age. Data represents mean ± SEM (*N* = 6–7), and different conditions were compared with a one-way ANOVA with Tukey’s multiple comparisons test: * *P* < 0.05; ** *P* < 0.01; *** *P* < 0.001 as compared with SOD1^WT^ mice. **e** Neuromuscular junctions (NMJs) innervation was scored in the gastrocnemius muscle. Data represents mean ± SEM (*N* = 5–6) and it was analysed with a two-tailed t-test. Representative images are shown of denervated and innervated NMJs in the gastrocnemius muscle that were stained with neurofilament-L (NfL) and α-bungarotoxin (BTX). Scale bar = 50 μm

We next examined electrophysiological and histological alterations. For this purpose, a group of SOD1^G93A^ EfnA5^+/+^ and SOD1^G93A^ EfnA5^+/−^ mice were followed from 60 days until 115 days for electrophysiological recordings. When mice became symptomatic, we analysed the number of motor neurons in the spinal cord and the percentage of innervated NMJs in the gastrocnemius muscle. The decrease of the CMAP amplitudes progressed similarly in both experimental groups until the last time point measured (Fig. 4a). The number of remaining motor neurons in the ventral horn of the lumbar spinal cord was comparable in both SOD1^G93A^ EfnA5^+/+^ and SOD1^G93A^ EfnA5^+/−^ mice (Fig. 4b). In agreement with this result a similar reduction could be observed in the expression levels of the neuron and motor neuron marker genes *Rbfox3* and *Chat* in the lumbar spinal cord of SOD1^G93A^ EfnA5^+/+^ and SOD1^G93A^ EfnA5^+/−^ mice as compared to SOD1^WT^ mice at the age of 130 days (Fig. 4c, d). The percentage of innervated NMJs in the gastrocnemius muscle was also similar in both genotypes (Fig. 4e).

### Ephrin-A5 reduction does not affect gene expression of glial markers nor the overall proteome

**Fig. 5:**
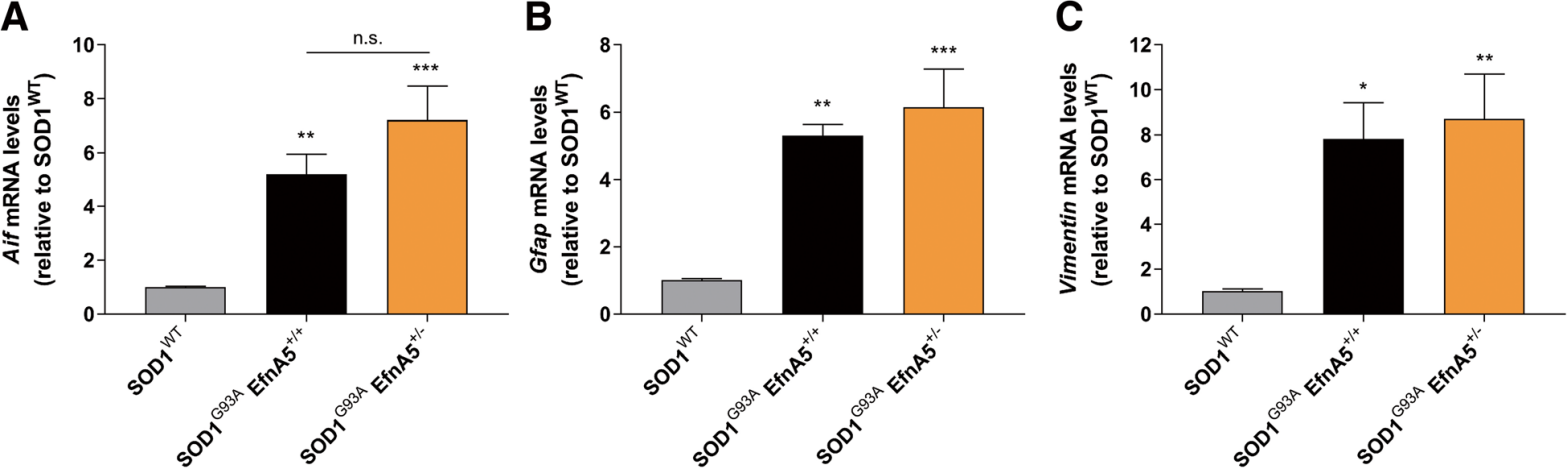
Reduction of efnA5 levels does not alter glial gene expression in symptomatic mice. Gene expression of several glial genes was quantified in the lumbar spinal cord of SOD1^WT^, SOD1^G93A^ EfnA5^+/+^ and SOD1^G93A^ EfnA5^+/−^ mice. **a** *Aif*, **b** *Gfap*, and **c** *Vimentin* levels were unaltered in SOD1^G93A^ EfnA5^+/−^ mice. Data represents mean ± SEM (*N* = 6–7), and different conditions were compared with a one-way ANOVA with Tukey’s multiple comparisons test: * *P* < 0.05; ** *P* < 0.01; *** *P* < 0.001 as compared with SOD1^WT^ mice

**Fig. 6:**
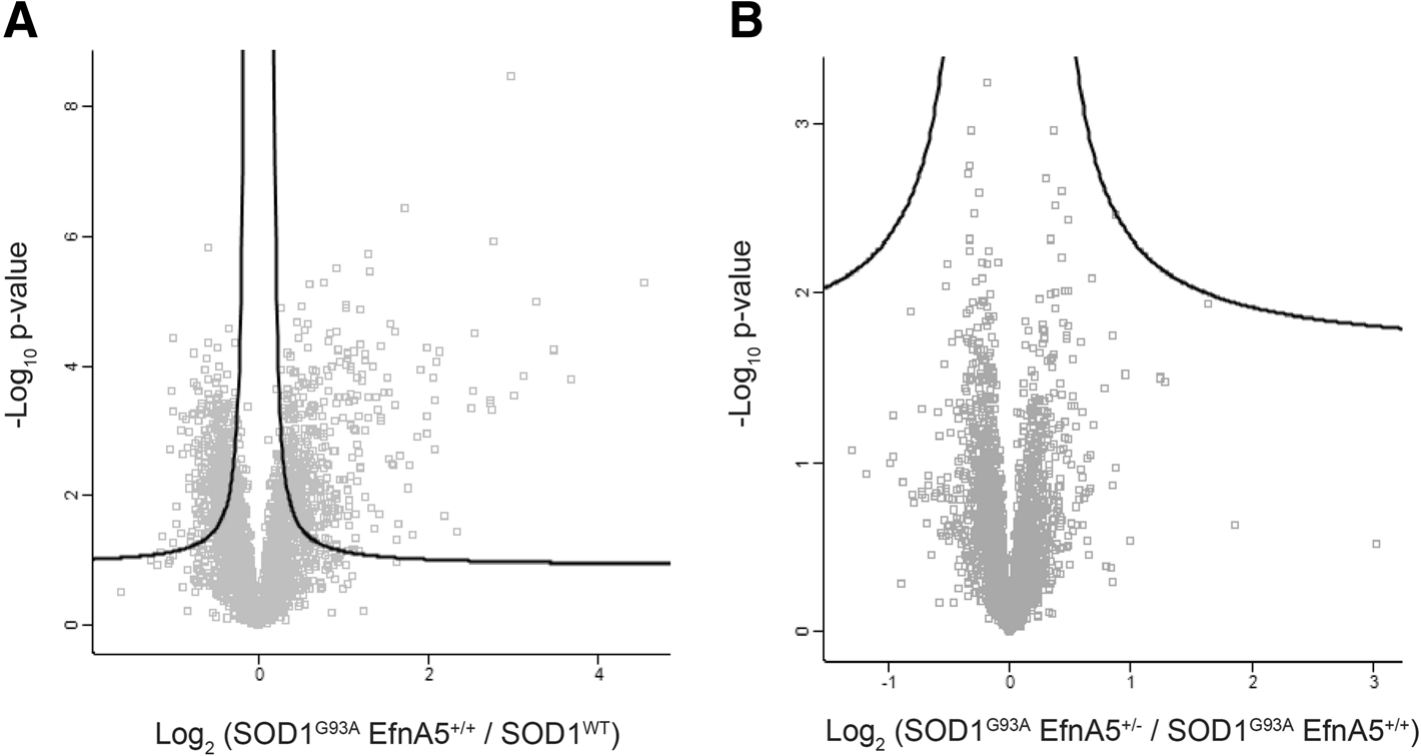
Whole proteome is not altered in the lumbar spinal cord of SOD1^G93A^ EfnA5^+/−^ compared to SOD1^G93A^ EfnA5^+/+^ mice at 130 days of age. Differences at a proteomic level were determined for **a** SOD1^G93A^ EfnA5^+/+^ versus SOD1^WT^, and for **b** SOD1^G93A^ EfnA5^+/−^ versus SOD1^G93A^ EfnA5^+/+^ mice. Data is represented in Volcano plots and the black lines indicate the significance level based on an FDR of 5% and S0 = 0.1 to correct for multiple testing

In ALS, non-cell autonomous mechanisms contribute to the selective motor neuron death [1, 19, 45]. Therefore, we analysed the expression in spinal cord tissue from mice at 130 days of age of several genes involved in glial function, which are altered in ALS, and compared their expression between SOD1^G93A^ EfnA5^+/+^ and SOD1^G93A^ EfnA5^+/−^ mice, to find a potential mechanism for the worsening of disease progression. The increase in the microglial marker *Aif* and the reactive astrocyte markers *Gfap* and *Vimentin* was analogous in SOD1^G93A^ EfnA5^+/+^ and SOD1^G93A^ EfnA5^+/−^ mice compared to SOD1^WT^ mice (Fig. 5a-c). Next, we performed a proteomic analysis in spinal cord samples from the same mice to detect alterations in any neuronal or glial molecular pathway in an unbiased way. We detected a total of 4065 proteins for the comparative analysis between the groups. Alterations in 817 proteins were altered in SOD1^G93A^ mice compared to SOD1^WT^ mice, but no differences could be detected between SOD1^G93A^ EfnA5^+/−^ and SOD1^G93A^ EfnA5^+/+^ mice (Fig. 6a, b).

### Similar re-innervation status after sciatic nerve injury upon efnA5 reduction

**Fig. 7:**
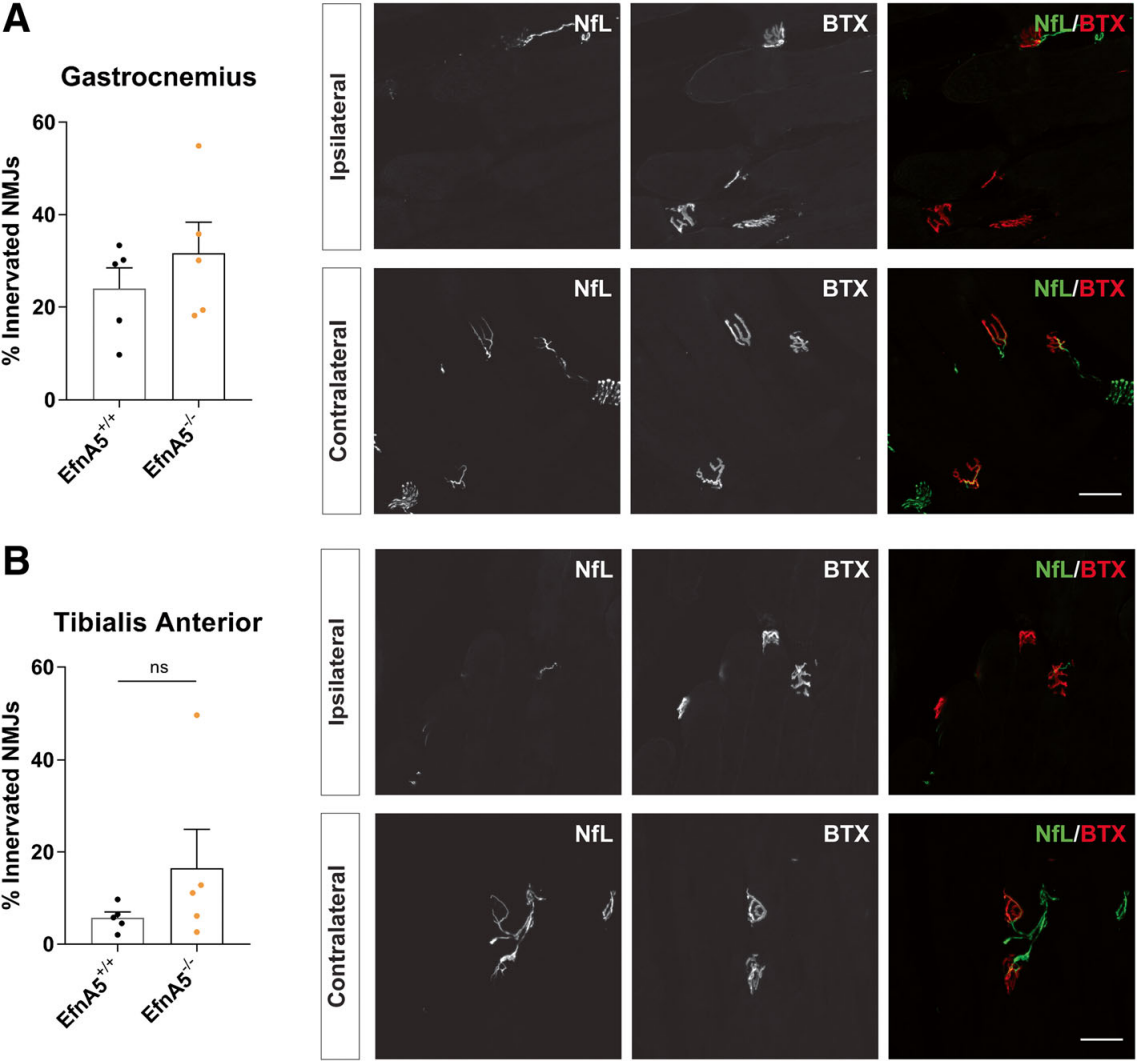
Axonal regeneration is not enhanced in EfnA5 knockout mice after sciatic nerve crush. Percentage of re-innervated neuromuscular junctions (NMJs) was determined in the **a** gastrocnemius and in the **b** tibialis anterior muscles in the ipsilateral and contralateral limbs of EfnA5^+/+^ and EfnA5^−/−^ mice 11 days after the crush (*N* = 5–5). Data represents mean ± SEM, and different conditions were compared with a two-tailed t-test. Representative images of ipsilateral and contralateral gastrocnemius and tibialis anterior muscles that were stained with neurofilament-L (NfL) and α-bungarotoxin (BTX) are shown. Scale bar = 50 μm

Although we did not find evidence for an increase in re-sprouting upon a 50% reduction of efnA5 expression in ALS mice, we examined whether a further reduction of efnA5 levels would affect the re-innervation potential in an acute nerve injury model. We crushed sciatic nerves of EfnA5^+/+^ and EfnA5^−/−^ mice (bred in a mixed background to prevent embryonic lethality) and evaluated re-innervation. We assessed re-innervation of NMJs 11 days after the surgery in two different muscles of the lower limb, the gastrocnemius and the tibialis anterior muscles. The ipsilateral limb showed a profound denervation and we did not observe any difference between EfnA5^+/+^ and EfnA5^−/−^ mice in the percentage of re-innervated NMJs (Fig. 7a, b), suggesting that efnA5 is not involved in axonal regeneration in acute neuronal crush injury.

### Lower levels of efnA5 in the cerebrospinal fluid of patients associates with faster disease progression

**Fig. 8:**
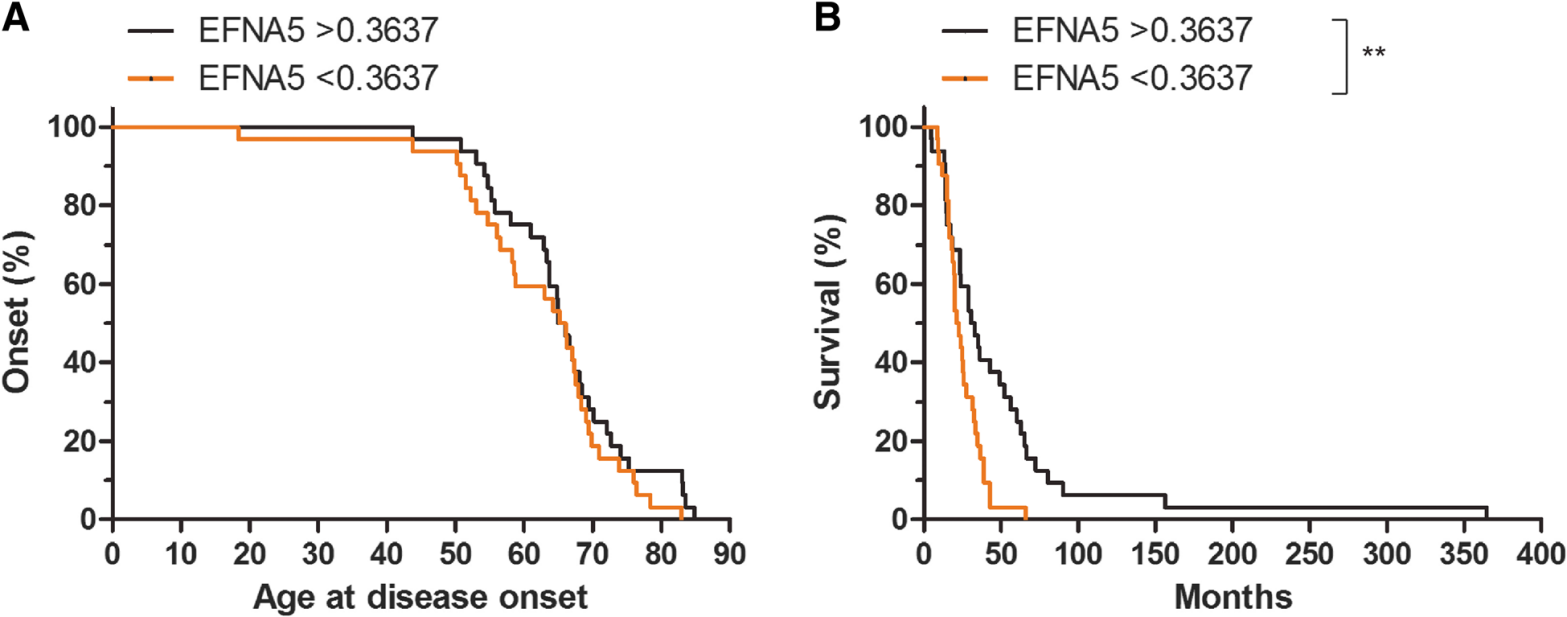
Disease progression is more severe in patients with lower CSF efnA5 protein levels. **a** Disease onset and **b** disease progression were determined in patients with CSF efnA5 levels above the median and in patients with CSF efnA5 levels below the median (*N* = 32). Differences were compared between the two genotypes with the Log-rank test: ** *p* < 0.01

To evaluate if the findings observed in the mouse model for ALS would translate to the phenotypic expression of the disease in humans we studied efnA5 protein in the CSF of ALS patients. We correlated efnA5 levels in the CSF with disease characteristics in ALS, by comparing patients with efnA5 protein expression below and above the median value in the ALS cohort. Disease onset was similar in these two subgroups, comparable to our observations in the mouse model. However, disease progression was more severe in patients with lower levels of efnA5 as reflected by shorter disease duration, also analogous to our results in the mouse model (Fig. 8a, b).

## Discussion

Previously we identified EphA4 as a disease modifier of ALS [42]. Here we studied the contribution of one EphA4 ligand, efnA5, in the disease progression of a mouse model of ALS. We observed that in the ventral horn of SOD1^G93A^ mouse spinal cord, efnA5 was predominantly present in almost all neurons and not in glial cells. Heterozygous deletion of efnA5 aggravated disease progression without affecting onset nor disease-specific glial and neuronal pathways. In an acute neuronal injury model the sprouting capacity of neurons did not improve in the absence of efnA5. Finally, patients with lower efnA5 levels had faster disease progression. Our findings indicate that reduced levels of efnA5 aggravate the clinical presentation of ALS and add to the evidence that altering ephrin signalling modifies the disease course in both mouse models and humans.

We selected efnA5 as target ligand, since in another neuronal injury model, stroke, evidence exists for upregulation of efnA5 in astrocytes, which reduced axonal regeneration after injury [33]. Moreover, the efnA5 gene contains conserved NF-κB binding sites, which makes it a direct downstream target gene of the pro-inflammatory NF-κB pathway [17]. The NF- κB pathway is activated in glial cells isolated from ALS patients and SOD1^G93A^ mice [13, 18], and there is substantial evidence on the contributing role for astrocytes, microglia and oligodendrocytes in the selective vulnerability of motor neurons in ALS [1, 19, 45]. We revealed predominant expression of efnA5 in neurons and minimally in glial cells in the adult mouse spinal cord, with no upregulation in mice overexpressing SOD1^G93A^. Several mechanisms could account for this lack of efnA5 expression in astrocytes. Despite the activation of the NF-κB pathway in astrocytes and other glial cells [13, 18], various gene expression regulatory elements in those specific cell-types could interfere with efnA5 upregulation. In addition, reactive astrocytes are also very heterogeneous, and depending on the insult, their transcriptional profile differs [24, 46]. As an example, stroke-induced reactive astrocytes upregulate neurotrophic factors and seem to acquire neuroprotective properties, whereas astrocytes activated by lipopolysaccharide enhance complement cascade genes, which can be harmful for synapses [24, 46]. ALS reactive astrocytes acquire a phenotype more similar to that upon induction by lipopolysaccharide, which may not be accompanied with efnA5 upregulation [18, 24].

Within the spinal cord different types of lower motor neurons exhibit diverse vulnerability. The largest fast-twitch fast-fatigable (FF) alpha-motor neurons are the first to degenerate in ALS mouse models and patients, and they are progressively followed by the smaller fast-twitch fatigue-resistant (FR) and later on by the slow (S) motor neurons [14, 35, 37]. In contrast, gamma-motor neurons, which are the smallest in size, are relatively preserved during the progression of the disease [22]. We previously identified EphA4 as a susceptibility factor contributing to the specific motor neuron vulnerability [42]. Similarly, we found in control mice the highest levels of efnA5 in the large motor neurons, and therefore we assessed the role of efnA5 levels in motor neuron vulnerability. At 135 days efnA5 expression in SOD1^G93A^ mice was reduced in these motor neurons compared to SOD1^WT^ mice, and by lowering efnA5 levels in the SOD1^G93A^ EfnA5^+/−^ mice disease progression was aggravated. Combining these findings we hypothesize that in contrast to EphA4, the vulnerability of motor neurons increases upon reduction of efnA5. However, the number of motor neurons did not differ in symptomatic SOD1^G93A^ mice with normal or reduced efnA5 levels. Moreover, we could not observe any difference in motor neuron molecular pathways that would indicate further neuronal dysfunction in mice with reduced efnA5 levels. Since onset was not altered in these mice but disease progression and survival were aggravated, reduced levels of efnA5 may disrupt neuronal integrity at very late stages of the disease. In addition, during ALS disease progression, remaining intact motor neurons have the capacity to re-sprout to compensate for the denervation of NMJs [14]. Interestingly, during development, neuronal efnA5 promotes the growth of LMCl motor axons that project to the dorsal limb [3, 23]. Therefore, we studied the sprouting capacity in an acute model for neuronal injury by crushing the sciatic nerve in control mice and in those lacking efnA5. However, we did not find differences in re-innervation in EfnA5^−/−^ mice. The recovery after the injury was similar in both genotypes, indicating that even a complete absence of efnA5 did not alter neuronal re-sprouting. In addition, innervation of NMJs at the gastrocnemius muscle at least just after onset was also not altered in SOD1^G93A^ mice with reduced efnA5 levels. These results suggest that, in contrast to developmental stages when efnA5 plays a role in axonal sprouting, in an adult-onset disease, efnA5 reduction does not alter retraction of motor axons from the muscles.

We initially considered efnA5 localized in motor neuron neighbouring cells such as astrocytes as a possible binding ligand of EphA4. However, we observed predominant neuronal efnA5 expression. Reducing efnA5 expression with 50% accelerated disease progression, whereas heterozygous deletion of EphA4 gene increased survival in SOD1^G93A^ mice [42]. Although highly speculative efnA5 and EphA4 expressed in the same motor neurons could establish inhibitory cis-binding. The absence of efnA5 may increase EphA4 signalling, resulting in shorter survival in mice. Inhibitory cis-binding has already been reported for efnA5 in retinal axons, where cis-binding of efnA5 with EphA3 desensitizes EphA3-mediated signalling [7]. Future research could be focussed on further elucidating this hypothesis and considering the implications for other ephrin members of the family. In addition, efnA5 is a ligand of most EphA receptors [30]. Since the interaction of Ephs and ephrins mediate intercellular communication [20, 34], another hypothesis would be that efnA5 mediates neuroprotection by binding in trans to other EphA receptors expressed in other cell types surrounding neurons. Further work will address whether these other Eph receptors might have a role in ALS. Interestingly, a neuroprotective response has been suggested for the neuronal EphB1, which interacts with the astrocytic ephrin-B1 to induce a neuroprotective phenotype in astrocytes [41].

In the pathophysiology of ALS various cell types acting in concert results in motor neuron death. Therefore, we studied alterations in various neuronal and glial markers in ALS mice with reduced efnA5 levels. However, we did not detect alterations in glial cell markers in the lumbar spinal cord of SOD1^G93A^ EfnA5^+/−^ mice when compared to SOD1^G93A^ EfnA5^+/+^ mice. In an unbiased proteomics approach, we found several up- and down-regulated proteins in late-symptomatic SOD1^G93A^ mice when compared to age-matched SOD1^WT^ mice, but we failed to detect any protein or molecular pathway in the spinal cord that was altered in SOD1^G93A^ EfnA5^+/−^ mice when compared to SOD1^G93A^ EfnA5^+/+^ mice, that could explain the observed modifying effect of efnA5. A limitation of our study is the fact that we performed a general efnA5 knockdown, instead of a nervous system specific knockdown. Further work and the use of conditional strategies to knockdown efnA5 in specific cell types and tissues will allow determination of cell types involved in the modifying role of efnA5 in ALS.

## Conclusions

Reducing levels of efnA5 aggravates disease progression without modifying the disease onset in both an ALS mouse model as well as humans. Since efnA5 expression is mainly neuronal we hypothesize a decrease in neuronal integrity in the later stages of the disease. Our future understanding of the exact mechanism that drives this accelerated disease phenotype, may gain more insights in the complexity of Eph-ephrin binding in motor neuron diseases.

## Additional file

Additional file 1: **Figure S1.** Reduction of efnA5 levels does not alter the numbers of motor neurons or innervated neuromuscular junctions in a non-ALS context. (a) Motor neurons were counted in the ventral horn of the lumbar spinal cord in EfnA5^+/+^, EfnA5^+/−^ and EfnA5^−/−^ mice. Data is represented as mean ± SEM (*N* = 4) and it was analysed with a two-way ANOVA. (b) Innervated neuromuscular junctions (NMJs) were scored in the gastrocnemius muscle of the same mice. Data represents mean ± SEM (*N* = 4) and it was analysed with a one-way ANOVA. (JPG 1900 kb)

## Data Availability

The datasets analysed during the current study are available from the corresponding author upon request.

## Abbreviations

ALS: Amyotrophic Lateral Sclerosis
BTX: α-bungarotoxin
CMAP: Compound muscle action potential
CSF: Cerebrospinal fluid
efnA5: Ephrin-A5
FF: Fast-twitch fast-fatigable
FR: Fast-twitch fatigue resistant
MRM: Multiple reaction monitoring
NfL: Neurofilament-L
NMJ: Neuromuscular junction
PFA: Paraformaldehyde
S: Slow
SOD1: Copper-zinc superoxide dismutase
