# Supplementary figures and images for: Reduction of ephrin-A5 aggravates disease progression in amyotrophic lateral sclerosis
Source: Acta Neuropathol Commun. 2019 Jul 12;7:114. doi: 10.1186/s40478-019-0759-6 (PMC6626434; doi:10.1186/s40478-019-0759-6)

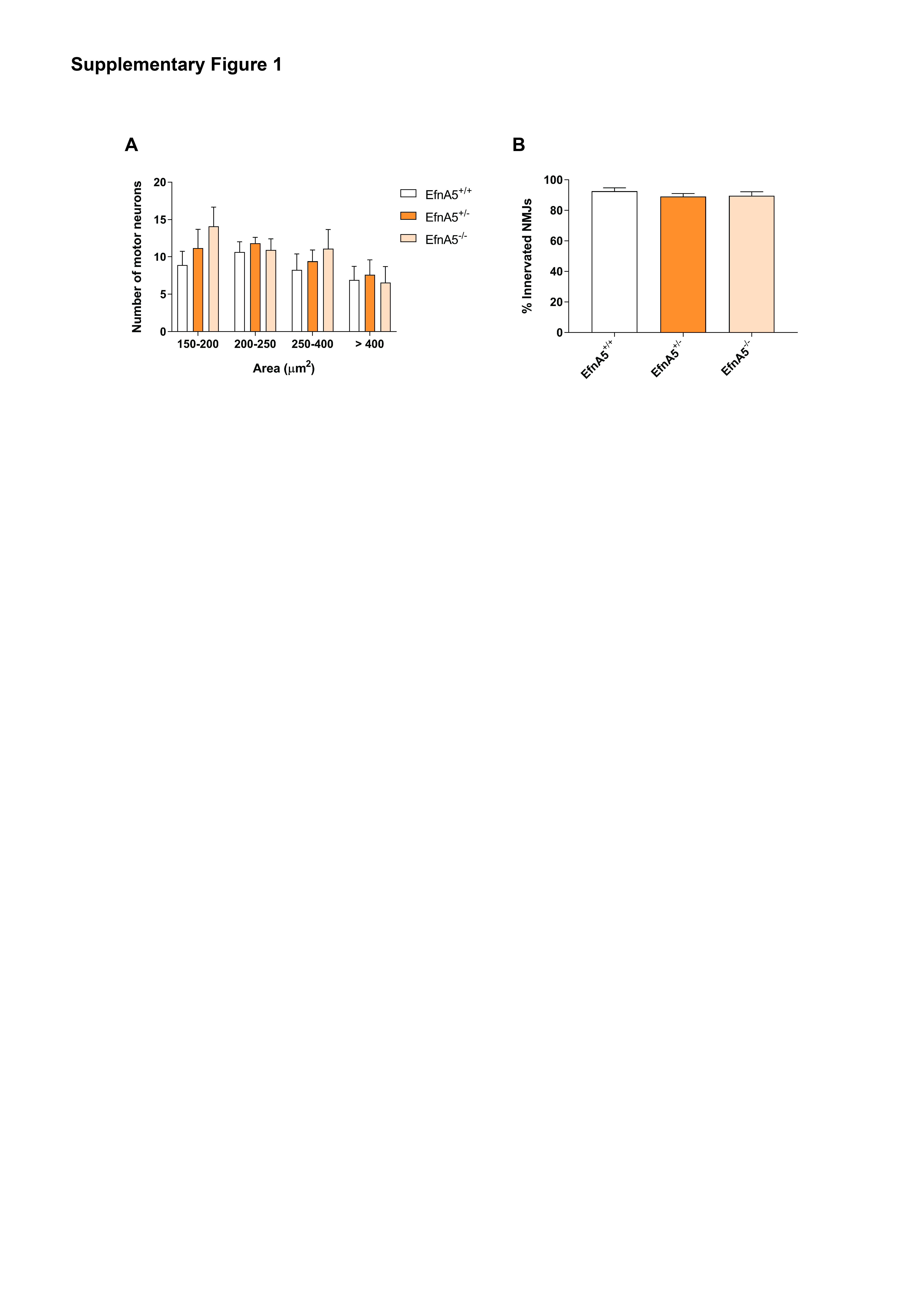

Supplement: Supplementary file 1 — Figure S1. Reduction of efnA5 levels does not alter the numbers of motor neurons or innervated neuromuscular junctions in a non-ALS context. (a) Motor neurons were counted in the ventral horn of the lumbar spinal cord in EfnA5+/+, EfnA5+/− and EfnA5−/− mice. Data is represented as mean ± SEM (N = 4) and it was analysed with a two-way ANOVA. (b) Innervated neuromuscular junctions (NMJs) were scored in the gastrocnemius muscle of the same mice. Data represents mean ± SEM (N = 4) and it was analysed with a one-way ANOVA. (JPG 1900 kb) [file 40478_2019_759_MOESM1_ESM.jpg]
